# Periscope: Pathology and Therapeutics

**Published:** 1818-10-01

**Authors:** 


### FOURTH DEPARTMENT.

THE

### Quarterly Periscope,

OR

SYNTHETICAL VIEW OF THE BRITISH JOURNALS,

WITH

*PRACTICAL COMMENTARIES.*

Nihil est aliud magnum quam multa minuta.

### I.

### *Pathology and Therapeutics.*

1. *Fever.* Mr. Kidd (Ed. Journ. 54.) has drawn an interesting sketch of the late typhous epidemic, as it ravaged the northern parts of Ireland, where the author then resided. Mr. K. traces the *predisposing* causes of this fatal scourge to the failure of the crops in the summer of 1816, owing to the excessive rains. Famine and wretchedness naturally followed, and fevers were the consquence. These were often *excited* by the habit of crowding into the close dirty rooms of the sick, and also by the ancient custom of sitting up at *wakes.*

Typhus having once gained a footing, would, under such circumstances, spread with great rapidity. It generally affected the upper classes more severely than the lower. In the symptoms there was nothing more than those modifications arising out of the habits, food, and disposition of the Irish. Mr. Kidd remarks, that the danger of the disease was by no means in proportion to the early violence of the symptoms. On the contrary, where the reaction was smart, and called for assistance, the issue was more fortunate. In fact, where the stage of what Dr. Armstrong justly denominates "*venous congestion,*" creeps on in a slow insidious manner, it requires to be very narrowly watched, for then the reactive powers of the system, or *vis medicatrix naturæ,* are very deficient, and some important organ may become so gorged as to destroy life.

Mr. Kidd's *methodus medendi* consisted principally in evacuation of the primæ viæ, by emetic-cathartic medicines at the beginning, and purgatives afterwards, with cool air, sponging the body, &c. No dissections could be obtained.

Mr. Archibald Robertson, surgeon of the Bellerophon, convict ship at Sheerness, has, in the same number of the *Ed. Journal,* made some observations on typhus, as it occurred in that ship. The cases could be traced to contagion.

"The symptoms exhibited the existence of increased vascular action and congestion, yielding in proportion as the mouth became affected by calomel, which was perseveringly exhibited in combination with opium and antimony."

### *Fever as connected with morbid Affection of the Brain and Spinal Marrow.*

Mr. James Sym, lately House Surgeon to the Glasgow Infirmary, has published (*Ed. Journ.* 55.) some interesting remarks on the connection in question. He judiciously observes, that the spontaneous accounts which patients give of their feelings in fever, almost uniformly contain, "head ache, and pain in the course of the spinal marrow, particularly at the lower part of it, where it splits into the cauda equina, and gives off the origins of the great nerves of the lower extremities." To this may be added, the sudden loss of power in all the muscles, with simultaneous derangement in the functions of all the viscera, leading to the conclusion, that the common source of those nerves, by which all are supplied with energy, must have ceased to perform its duty in a healthy manner.

The principal *post mortem* appearances in the Glasgow fever were, turgidity of the cerebral vessels, especially the venous branches; serum in the angles between the sides of the veins and the convolutions of the brain; *centrum ovale* dotted with small drops of blood; water in the lateral ventricles, and in the hollow of the occiput, seemingly from the spinal canal. When the body was placed prone, a large quantity always flowed from the theca vertebralis; when the canal of the third and fourth lumbar vertebræ

was laid open, by sawing through the transverse processes, and removing the posterior part of the tube, the dura mater was found distended with fluid, and the cauda equina was found floating amongst serum. These morbid appearances justify the conclusion that, in most instances, there was inflammatory action along the brain and spinal marrow. The paper is very creditable to Mr. Sym.

2. *Tic Douloureux.* Mr. Kerrison relates an interesting case of this terrible complaint (*Med. Repos. April*) evidently complicated with gouty affection and herpetic eruption, in which all the usual remedies, and even the division of the nerves, were tried in vain. Calomel and opium gave a temporary relief. At length the cinchona, in large doses, was resolutely administered, and a cure took place. We are by no means convinced that this cure was entirely the result of remedies; since, in similar habits, we have seen the disease run a long course, like its parent Gout, and then suddenly disappear *spontaneously*.

3. *Diabetes.* Dr. Trotter has called the attention of the faculty (*London Med. Journal*, 231) to the use of calcined magnesia in diabetes, two cases of which he has cured by this remedy. He began with half a drachm, mixed with a few grains of bark or columbo, twice a day, gradually increasing the dose till the patient took upwards of four scruples in the same period. Although purging followed, the strength increased; the voracious appetite declined, and the skin resumed its natural functions. The simplicity of the remedy and the fatality of the disease render Dr. Trotter's paper an object of trial and attention.

4. *Dropsical Affections.* Dr. Abercrombie (*Ed. Journ.* 54) has described a dropsical affection of the chest, depending, he conceives, on an inflammatory state of the lungs.

The disease comes on suddenly, affecting persons in the vigour of life, and is usually ascribed to exposure to cold after the body has been heated. It commences with oppressed and uneasy breathing, which is quickly succeeded by dropsical swellings, first in the face, and then in other parts of the body. The urine is scanty and high coloured; in some cases coagulable, in others shewing no trace of albumen. If left alone, the swellings increase; the breathing becomes more difficult, and death may take place at the end of a few days or a few weeks.

*Treatment.* Early and free blood-letting, till the pulmonary symptoms are relieved. If the dropsical swellings do not disappear, diuretics are to be employed.

5. *Purulent Expectoration without Ulceration or Abscess.* Dr: George Pearson, the distinguished Physician of St. George's Hospital, has (*Ed. Journ.* 54) related a remarkable instance of this kind, where, for more than a year, a large quantity, sometimes a pint daily, of matter, in every respect resembling that in confirmed phthisis, was spat up. The patient died, and on cutting into the lungs no vomicæ or ulceration were found. There was, however, dropsical effusion in the chest. We have seen several instances of this kind ourselves. We believe it is still denied that purulent matter can come from unabraded surfaces; but if the *puriloid secretion* cannot be distinguished from pus, it is of little consequence to agitate the question.

6. *Blood-letting from the hæmorrhoidal Veins.* Dr. Brown (*Ed. Journ.* 54) has drawn the attention of the Faculty to this practice, so common on the continent. The paper on the *hæmorrhoidal movement,* inserted in the present number of this Journal, will render all observations on Dr. Brown's hints unnecessary. Leeches to the verge of the anus are certainly an important mean of mitigating various abdominal diseases connected with venous plethora of the portal circle.

7. *Tar Vapour in Spasmodic Asthma.* Mr. Wansbrough (*Med. Repos. No.* 54) relates a case of this distressing complaint, which *appeared* to give way suddenly to the vapour of tar. Into an earthen pipkin a pound of liquid tar was poured, together with an ounce of nitre. The pipkin was then placed on the fire until the tar boiled. In this state it was placed in the centre of the room on a table. As soon as the fumes reached the patient, she made signs to bring the apparatus nearer to her, " and actually drew it herself, in a few minutes, directly under her nose." In half an hour she exclaimed, " *O! Sir, I am in Heaven!*" We apprehend that this case is related with rather too much *stage effect,* to hold forth very confident hopes. It is, however, deserving of further consideration.

8. *Chronic Inflammation of the Brain and its Membranes.* Dr. Abercrombie has favoured the medical world with a very valuable paper on the above important subject, in the 55th No. of the *Ed. Med. and Surg. Journal,* accompanied by a great variety of cases in illustration. The symptoms of chronic inflammation of the brain, he thinks, may be referred to four classes.

Class I. This chiefly appertains to children, and is usually preceded by languor and peevishness, followed by fever, which is sometimes ushered in by rigors. Oppression, disinclination to motion, severe pain in some part of the head, flushing of the face, and intolerantia lucis succeed; occasional vomiting; pain sometimes extends from the head along the neck, and even to the shoulders, or other parts of the body; pupil usually contracted; eye sometimes suffused; tongue white, moist, sometimes quite clean; sleep disturbed by frightful starting dreams, with grinding of the teeth. Bowels generally obstinate, but sometimes natural, or loose. After some days, delirium appears, or forgetfulness and misnaming of things. These are followed by tendency to sleep and coma. While these changes are going on, the pulse, which was at first frequent, usually falls to the natural standard, or below it; the pain becomes less violent; the eye loses its sensibility, becoming dull and vacant, often with squinting and double vision, succeeded by blindness and dilated pupil. The pulse now begins to rise to extreme frequency, often as high as 200 in the minute, with extreme irregularity. Coma and paralysis generally terminate the scene.

Class II. This mostly appertains to young persons towards the age of puberty. It begins with slight head-ache, general uneasiness, disturbed sleep, impaired appetite, foul tongue, pulse from 96 to 100. These symptoms are ameliorated by slight means, and every thing appears to be going on well; but presently there is an exasperation, and more active practice is employed, and again there is an appearance of amendment, but still some head-ache and quickness of pulse remain. Amid these exacerbations and remissions, eight or ten days pass before the disease assumes a decided character. Perhaps, about the 12th or 14th day, the pulse suddenly falls to the natural standard, or below it, while the head-ache is increased, with a tendency to stupor, marking decidedly a most dangerous affection. The patient now lies for several days in a state of considerable stupor, sometimes with convulsions, often with squinting and double vision. The pulse then begins to rise again, and, about this time, there is frequently a deceitful interval of apparent amendment; but he soon relapses into perfect coma, and dies in three or four days. The duration of the complaint is uncertain; it may be drawn out to five or six weeks, or it may be fatal in two or three.

Class III. This is peculiar to adults, and begins with violent head-ache and fever; the pulse not indicating any thing particular; face, in some cases flushed, in others, pale. The pain of the head is usually deep seated, frequently seeming to shoot from temple to temple. There is a look of much oppression, sometimes vomiting. Delirium sometimes appears early, varying in degree from day to day, until, after six or seven days, it passes into fatal coma; the pulse all along from 70 to 80. In some cases, vision is not affected; in others, squinting and double vision occur. In every case there is more or less delirium, but often slight and transient.

Cass IV. Another and very frequent form of the disease is ushered in by a violent attack of convulsion, in some cases followed immediately by coma, which, in a few days, is fatal.

Our author does not consider chronic inflammation of the brain as always idiopathic. It often supervenes on other diseases, as *fever,* scarlatina, measles, pneumonia, &c.

Its *terminations* may be fatal in the inflammatory stage; or by serous effusion, suppuration, or a peculiar destruction or disorganization of the central parts of the brain; the fornix, septum lucidum, or white medullary matter lining the ventricles. This consists of those parts being broken down into a white, soft, pulpy mass, retaining their natural colour, but losing their figure and consistence. It is often combined with the deposition of coagulable lymph, in the immediate vicinity of the parts affected, or with suppuration in other parts of the brain, or serous effusions in the ventricles. Another mode of termination is in the thickening of the membranes, contraction of the sinuses, caries of the bones, and other affections of the external parts.

In the pathology of this affection, Dr. A. justly thinks, that too much attention has been paid to the serous effusion, or hydrocephalus, as if this alone constituted the disease, though it is probably only one of the many terminations of chronic inflammation of the brain.

Dr. A. next introduces a great number of valuable and interesting cases illustrative of the foregoing positions, followed by a train of judicious general remarks; for which we refer to the paper itself. Dr. Abercrombie is unquestionably a man of acute observation, sound judgment, and unwearied assiduity. These are the kind of men who are likely to promote the march of medical science.

9. *Intermitting Rheumatism.* Mr. N. Rumsey (*Ed. Journ.* 55) relates several instances where rheumatism affected, in a periodical type, certain internal tissues, especially the membranes of the abdominal muscles, producing sympathetically, flatus and distention in the bowels, with febrile symptoms generally; these phenomena were removed by the exhibition of bark. We have seen numerous instances of this kind. In fact, both gout and rheumatism often lie masked in the shape of anomalous pains and morbid feelings of parts where they are little suspected.

10. *Hemicrania cured by Leeches.* Mr. D. R. Robinson relates (Ed. Journal, 55) a case of severe hemicrania, particularly affecting the first and second branches of the fifth pair, which resisted all means that were tried, till twelve leeches were applied to the temples, which were repeated three or four times afterwards, but in greater number. The bowels were kept open in the mean while.

We are persuaded, that local abstractions of blood, together with calomel and opium, are the only means to be relied on in such cases.

11. *Enlarged Ovarium.* Mr. Edwards of Bath, in the same Journal, relates a complicated case of [supposed] dropsical ovarium, with mental derangement, anasarca, &c. We just as much believe that Mr. Edwards cured ovarian dropsy, as that he cured "a dropsical state of the brain and *effusion into the ventricles.*" No such good luck has ever turned up in our practice, or in the practice of any one with whom we are acquainted.

